# Modulation of the Tumor Microenvironment for Cancer Treatment: A Biomaterials Approach

**DOI:** 10.3390/jfb6010081

**Published:** 2015-02-17

**Authors:** Isaac M. Adjei, Sharma Blanka

**Affiliations:** Department of Biomedical Engineering, University of Florida, Gainesville, FL 32611, USA; E-Mail: adjeii@ufl.edu

**Keywords:** 3D tumor model, mesenchymal stem cells, nanocarriers, immune reprogramming, tumor stroma

## Abstract

Tumors are complex tissues that consist of stromal cells, such as fibroblasts, immune cells and mesenchymal stem cells, as well as non-cellular components, in addition to neoplastic cells. Increasingly, there is evidence to suggest that these non-neoplastic cell components support cancer initiation, progression and metastasis and that their ablation or reprogramming can inhibit tumor growth. Our understanding of the activities of different parts of the tumor stroma in advancing cancer has been improved by the use of scaffold and matrix-based 3D systems originally developed for regenerative medicine. Additionally, drug delivery systems made from synthetic and natural biomaterials deliver drugs to kill stromal cells or reprogram the microenvironment for tumor inhibition. In this article, we review the impact of 3D tumor models in increasing our understanding of tumorigenesis. We also discuss how different drug delivery systems aid in the reprogramming of tumor stroma for cancer treatment.

## 1. Introduction

As our understanding of cancer pathogenesis progresses, increasing attention is being placed on the tumor microenvironment, or “tumor stroma”, that provides the conditions permissive for the growth and progression of malignant cells [1,2]. A better understanding of the tumor microenvironment and how to manipulate it into one that is less or non-permissive to tumor development are central to emerging cancer therapies [3]. The quest to advance our knowledge of tumor biology has been facilitated by biomaterials and fabrication techniques that allow us to mimic various characteristics of tumors *in vitro*. Most notable is the development of 3D extracellular matrix analogs to study tumor development in a physiologically relevant manner [4]. The biomaterials provide media into which various stromal elements can be incorporated to identify new disease mechanisms and targets for treatment [5,6]. Biomaterials also serve as tools in treating cancers and have been developed into drug/gene delivery systems, such as nanoparticles (NPs), to target cancer cells in the tumor, and can be re-engineered to target stromal cells, as well [7,8]. This review provides an overview of the cells that contribute to the tumor stroma, the biomaterials that have been used to develop 3D tumor models and to study the tumor microenvironment and biomaterials used to deliver therapeutics aimed at disrupting the tumor microenvironment.

## 2. Microenvironment in Cancer Initiation and Progression

Tumors consist not only of the malignant cancer cells, but also of stromal cells that support the tumor microenvironment. These include fibroblasts and immune cells [9,10], as well as endothelial cells and smooth muscle cells that form blood vessels and provide nourishment to the tumor [11]. In addition to the cellular component, the extracellular matrix (ECM) and secreted extracellular molecules act in autocrine and/or paracrine manners to support/sustain tumor development. 

By themselves, stromal cells are not malignant and function to maintain normal tissue structure and function. However, through intercellular interactions or paracrine secretions by cancer cells, normal stromal cells acquire abnormal phenotypes that support cancer cell growth and tumor progression [12,13]. In their dysfunctional state, fibroblast and immune cells produce chemokines and growth factors that stimulate cancer cell growth and invasion and can recruit other cells, including mesenchymal stem cells (MSCs) that replenish cells in the tumor [14]. 

As we learn more about cancers, the important role of the tumor stroma in tumor progression is being realized. It is necessary to learn more about the relationships between the different components of tumor stroma and cancer cells and how they relate to tumor progression and metastasis, in order to develop better strategies to treat the disease. Here, we highlight some of the roles of tumor stromal cells, particularly those that have been exploited for cancer therapies. The biology of the tumor stroma and the role of the different stromal cell populations are well documented and summarized in several reviews [15,16,17,18].

### 2.1. Role of Different Cell Populations

Solid tumor contains non-malignant cell types that act in different capacities to support tumor growth and metastasis (Table 1). 

**Table 1:** Cellular components of tumor stroma.

Lineage	Role in tumorigenesis
Tumor-associated macrophages	Immunosuppression; produce cytokines and growth factors Tumor remodeling; secrete matrix metalloproteinases (MMPs) and urokinase-type plasminogen activator (uPA)
Neutrophils	Produce cytokines and reactive oxygen species
Treg cells	Immunosuppression; secrete TGF-β and IL-10 that inhibit the antitumor activity of cytotoxic T-cells and natural killer cells
Th cells	Production of cytokines that induce immunosuppression
B-cells	Production of cytokines and activation of mast cells
Mesenchymal stem cells	Produce cytokines that promote tumor invasiveness and metastasis; Replenish cancer cells
Tumor-associated fibroblasts	Secrete MMPs involved in tumor remodeling; Produce vascular endothelial growth factor (VEGF) that induce angiogenesis
Vascular endothelial cells	Form blood vessels that support tumor growth and metastasis

#### 2.1.1. Fibroblasts

Fibroblasts comprise the predominant cells in tissue stroma and produce extracellular matrix (ECM). In normal tissues, fibroblasts produce different collagen subtypes and fibronectin, which contribute to the tissue basement membrane. They continuously remodel the ECM through matrix metalloproteinases (MMPs) and other proteases and are responsible for the overall architecture of tissues [19]. In addition to these functions, fibroblasts secrete a family of heparin-binding proteins (fibroblast growth factors) that activate the RAS-MAP kinase and PI3 kinase/AKT pathways, thereby promoting cell proliferation and survival [20,21]. 

Cancer-associated fibroblasts (CAFs) have phenotypes that are significantly different from normal fibroblasts. Like fibroblasts in wounds, CAFs express α-smooth muscle actin and splice variants of fibronectin, which are involved in cell contraction and wound closure [22]. However, unlike fibroblasts in wounds, CAFs do not revert back to their inactivated state, or undergo apoptosis. CAFs overexpress platelet-derived growth factor (PDGF) receptor-β that promotes their own cell proliferation and survival [23]. CAFs also secrete growth factors (transforming growth factor β, hepatocyte growth factor, insulin-like growth factor 1/2) and chemokines (monocyte chemotactic protein 1 and interleukin 1) that facilitate proliferation and invasion of cancer cells [24,25]. In addition, CAFs produce MMPs, mostly MMP-9 and MMP-2, and other matrix-modifying enzymes, including urokinase-type plasminogen activator (uPA), that degrade the ECM and support tumor invasion and metastasis [26]. 

Changes in collagen metabolism as part of tumor remodeling also affect tumor progression, and this is mediated by CAFs. A rise in collagen density corresponds with an increased rate of tumor initiation and invasion through the pro-tumorigenic activity of TGF-β [27,28]. Increased collagen makes tissues stiffer, modifies focal adhesions for cells and activates Rho-GTPase signaling, which results in cell proliferation [29]. 

#### 2.1.2. Immune Cells

Tumors have been described as persistent wounds that will not heal and, consequently, are infiltrated by immune cells, mostly macrophages and T-lymphocytes, that seek to kill the cancer cells. However, cytokines produced within the tumor subvert their actions and make them immune incompetent [18]. 

The role of tumor-associated macrophages (TAMs) in cancer is a highly debated subject. In non-small cell lung (NSCL) and thyroid cancers, high TAM density was associated with poor survival [30,31]. However, in some cervical cancers, TAM density was associated with better prognosis [32]. The role of TAMs in tumors is not clear and can vary even in the same type of cancer. For example, there are conflicting reports about the role of TAMs in NSCLC regarding whether they are supportive or inhibitive of tumor growth [30,33,34]. TAMs support or inhibition of tumor progression is dictated by their phenotype. Monocytes recruited into the tumor microenvironment from circulation can differentiate into one of two lineages of mature macrophages depending on the cytokine milieu in the tumor. Macrophages activated classically by interferon gamma (IFN-γ) are termed M1 and are pro-inflammatory. M1 macrophages are phagocytotic, cytotoxic and inhibit tumor progression [35]. All other macrophages whose activation is initiated by other inductions, such as IL-4 and IL-13, are termed M2. The M2 macrophages promote tissue repair, angiogenesis and produce cytokines that suppress the adaptive immune system, thereby supporting tumor progression [35,36]. 

Within tumors, the cytotoxic activity of M1 macrophages is inhibited by IL-4, IL-6, TGF-b1 and myocardiac depression factor secreted by cancer cells. On the other hand, inflammation is promoted by activation of NF-κB-associated pathways in the macrophages [37,38] through the action of IL-12 and TNF-α secreted by cancer cells [39]. The M2 TAM phenotype supports angiogenesis in tumors, by secreting VEGF and IL-8, which stimulate the proliferation of tumor-associated endothelial cells [40]. In addition to secreting cytokines and promoting angiogenesis, TAMs also have roles in ECM remodeling and produce MMPs, uPA and uPA receptor, which facilitate ECM degradation in corroboration with cancer-associated fibroblasts [41,42]. The role of TAMs in tumor initiation and progression is diverse and complex, and a complete review is beyond the scope of this article, but can be found in other review articles [43,44]. 

T-cells as part of the adaptive immune system should, in principle, function to rid the body of tumor cells. However, due to CCL2 (monocyte chemoattractant protein 1, MCP1) produced by cancer cells and tumor stromal cells, namely TAMs and CAFs, T-cells that infiltrate tumors become immunosuppressive CD4+ CD25+ T regulatory leucocytes (Tregs) [45]. Within tumors, Tregs produce TGF-β and IL-10, which contribute to an immunosuppressive environment through the inhibition of cytotoxic T-cells and natural killer cells. Tregs also bind to IL-2, making the cytokine unavailable in the tumor microenvironment to activate other immune cells [46]. Please make the style 

#### 2.1.3. Stem Cells

Tumor formation requires self-renewal of cancer cells. In the hierarchical models of tumor progression, this property is provided by a subpopulation of cancer cells, termed cancer stem cells (CSCs) [47]. CSCs can arise from normal tissue resident stem cells through oncogenic mutations or may be normal somatic cells that acquire oncogenic mutations that prevent them from entering post-mitotic differentiation states [48]. Stem cells are also recruited from the circulation and/or from nearby tissues into the tumor stroma [15]. Infiltration of tumors by circulating MSCs is enhanced by CXCR4, CXCR12 and CCL2 secreted by cancer cells [49,50]. In the tumor stroma, the role of MSCs remains unclear. Recruited MSCs can produce cytokines, principally CCL5, which enhance the migration, invasion and metastasis of cancer cells [49,51]. Other studies have shown that MSCs inhibit Akt protein kinase activity and downregulate Bcl-2 in cancer cells, which induce apoptosis [52,53]. The effect of MSCs on tumor growth can be found in several reviews [54,55]. Despite these contradictory findings, the tumor tropism properties of MSCs have led to therapeutic strategies aimed at using them as vehicles for anti-cancer drug/gene delivery [56,57] .

#### 2.1.4. Vascular Endothelial Cells

Angiogenesis in solid tumors is necessary to support the nutrient and oxygen requirements of the growing tumor [58]. This is facilitated by different tumor-associated cells, including vascular endothelial cells, which line the lumen of the blood vessel. Tumor vascular endothelial cells differ from normal endothelial cells in that they are abnormal in shape, highly fenestrated, have high motility [59] and form leaky blood vessels [60] that are routes for cancer cells to enter circulation to initiate metastasis. The highly fenestrated vessels also limit the accumulation of small molecules drugs in tumors, as they are cleared from the tumor environment. However, drug delivery systems, like nanoparticles and microparticles, utilize this enhanced permeability of tumor vasculature to localize within tumors [61]. Because of their role in disease progression, vascular endothelial cells in tumors have been the target of several cancer therapies with the rationale that cutting off blood supply will inhibit tumor growth [62]. We discuss this further in Section 4.1 of the review. 

## 3. Modeling Cancer Progression Using Tissue Engineering Concepts

**Figure 1:**
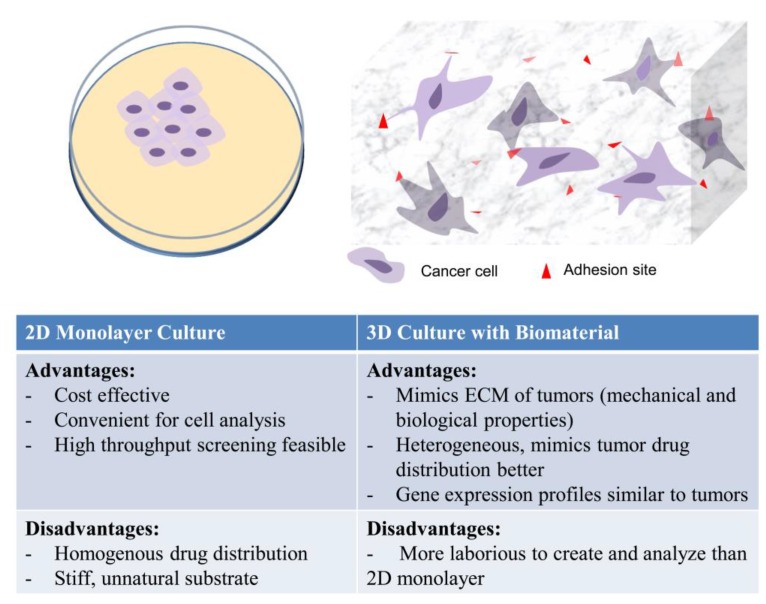
Strengths and weaknesses of 2D and 3D *in vitro* culture. Cells in 2D monolayer cultures lose their morphology and polarity, while cells in 3D matrices retain their morphology.

The study of human cancer biology, as well as the development and testing of anti-cancer drugs, typically begins with *in vitro* culture of cancer cells in Petri dishes. Two-dimensional monolayer cell cultures were used in early efforts to understand the interactions between cancer cells and tumor stromal cells and how these interactions influenced the disease process. However, these 2D systems have poor resemblance to the 3D *in vivo* tumor environment and often have little value in predicting the clinical efficacy of therapies [63]. For example, cancer cells in 2D demonstrate uniform growth, with most cells at the same cell cycle stage, unlike cancer cells *in vivo*, which are at different stages of the cell cycle. They also do not capture the phenotypic heterogeneity in terms of gene expression and differentiation in tumors [64,65]. To overcome some of these shortfalls, 3D culture systems have been employed, in which cancer cells lose polarity and form cell aggregates, thereby accounting for the tumor architecture that is absent in 2D cultures (Figure 1) [3,66,67]. These characteristics make 3D models physiologically relevant systems for the study of tumor dynamics and response to therapies [4,68]. 

### In Vitro 3D Models in Studying Cancer Biology

Much of the early work developing 3D cultures used Matrigel, which is a biologically-derived ECM now commonly used as a substrate in cancer cell migration and invasion assays. However, as with most purely natural ECM materials, there is little control over the physical and biological properties of Matrigel. Therefore, systematic studies of various physical, biological and mechanical elements of the tumor microenvironment are difficult to achieve [69].

To study these characteristics, biomaterials and 3D culture systems initially developed in the tissue engineering and regenerative medicine fields have been adopted to develop better *in vitro* models that recapitulate *in vivo* tumor characteristics in a controllable manner. This permits the evaluation of tumor architecture and stiffness on disease progression, as well as interactions between the different components of the tumor [6,70,71,72]. Cancer cells grown in 3D make physiologically relevant cell-cell and cell-ECM interactions, which can result in gene expression that is similar to that of actual tumors [73]. Cancer cells in 3D models also exhibit the slow cell proliferation and resistance to chemo- and radiation therapy observed in tumors *in vivo* [71]. The differences in architecture and gene expression of 3D models to 2D cultures may explain why they consistently produce IC_50_ to drugs that are several folds higher than that observed in cancer cells in 2D monolayers [74].

The mechanical properties of tumors, such as stiffness, can contribute to the progression of cancer from benign to malignant. High tumor stiffness promotes the metastatic transformation of cancer cells [75,76] and can be an indication of the invasiveness of the tumor [77]. Because the mechanical properties of the scaffolds used in 3D tumor models can be tuned, they can be designed to mimic stiffness and other mechanical properties of tumors in order to understand their impact on tumor invasiveness and metastatic potential. Poly(ethylene glycol) (PEG) hydrogel arrays with elastic moduli from 0.34 to 17 kPa, formed by modulating the concentrations of both the PEG ortho-nitrobenzyl backbone and the thiol-PEG-thiol crosslinker, demonstrated that cells grown in hydrogels with higher elastic moduli migrated faster than cells in hydrogels with lower elastic moduli [78]. Carey *et al.* also recently demonstrated, using collagen gels, that the microarchitecture within tumors affects the invasiveness of breast cancer cells. Cells cultured in fibrillar collagen gels with large collagen fibers (5.8 µm) were more mobile than cells grown in gels with small collagen fibers (2.0 µm) [79]. Taken together, these studies show that it is necessary to consider both the overall bulk characteristics and microarchitecture of scaffolds when studying their effect on tumor cells. 

Multicellular tumor spheroids (MCTS) are the most common 3D cultures used in cancer biology. Spheroids can be formed by different techniques, including the hanging drop technique, which is automated for high throughput screening to determine drug efficacy and toxicity [80]. Unfortunately, standard methods for making spheroids do not produce samples that are consistent in terms of size and cell numbers. To address these issues, various techniques have been developed. One such technique utilized magnetic fields. In these systems, cell-adhesive peptide modified magnetic nanoparticles are first incubated with the cells, which are subsequently manipulated with an external magnetic field to produce millimeter-sized 3D cultures [81,82]. Spheroids created with these and other techniques are held together mostly through cell-cell interactions. Signaling pathways involved in cell-cell interactions have been studied in high throughput screening using small hairpin RNAs to identify genes that have a role in these interactions [83]. In addition, spheroids show that conformation of cell surface proteins is affected by the context in which they are presented. Breast cancer cells, for example, present human epidermal growth factor receptor-2 (HER2) as heterodimers when in 2D culture, but as homodimers in 3D culture, which results in different responses to trastuzumab [84,85]. 

*In vitro* 3D models have facilitated progress in the understanding of the different stages of cancer progression. Several biomaterials, particularly hydrogels, which have high tissue-like water content and tunable physical and mechanical properties, have been used to model different stages of cancer. Hydrogel scaffolds made from collagen type I and cultured with MDA-MB-231 breast cancer cells [86] generate oxygen and nutrient tension across different depths of the scaffold structure and cause necrosis in deep layers of the scaffold that is reminiscent of the pre-vascularized stage of solid tumor progression. For angiogenesis, bilayered 3D hyaluronan hydrogels formed by thiol-acrylate crosslinking significantly increase the expression of vascular endothelial growth factor-165 (VEGF_165_) and interleukin-8 (IL-8), both of which are involved in angiogenesis [87]. Alginate hydrogels modified with RGD peptides, on the other hand, have helped demonstrate that the interactions of cancer cells with α5β1 integrins in a three-dimensional tumor microenvironment is important in the regulation and the secretion of VEGF and IL-8 and, consequently, angiogenesis. These alginate scaffolds also demonstrate that hypoxic conditions increase VEGF secretion, but not the secretion of IL-8 [88]. 

Metastasis has generally been studied using animal models. However, there are efforts to develop *in vitro* models that recapitulate *in vivo* metastasis or metastatic niches, in order to better delineate the different stages of the metastasis process [70]. To this end, different biomaterials and bio-fabrication methods are in development that allows biologists to study different aspects of metastasis. For example, invasion of breast cancer cells (MDA-MB-231) into surrounding tissues has been studied with 3D fibroin matrices and has been shown to involve tissue ECM degradation by MMP-9 [89].

The tropism of cancer cells to different tissues during metastasis has also been investigated using biomaterials. Solid poly(lactic-co-glycolic acid) (PLGA) scaffolds mineralized with hydroxyapatite nanoparticles (NPs), for example, have been developed as a bone mimic [90]. Breast cancer (MDA-MB-231) cells that metastasize to bone have better adhesion to this bone mimic, demonstrate increased proliferation and secrete high levels of IL-8 (encourages bone resorption) than cells that do not metastasize to bone [90]. Models such as these help identify cell surface proteins that increase the propensity of certain cancer types to metastasize to particular tissues. When these models are used in conjunction with 3D microfluidic models, extravasation of cancer cells into tissues from circulation can be studied. One such model utilizes a 3D ECM-hollow endothelial channel that allows the monitoring of cancer cell movement across the lumen [91]. Systems like this can be enhanced further by the introduction of different stromal cells or paracrine signaling to elucidate their role in cancer cell extravasation. 

The development of 3D culture systems has opened new opportunities in the quest to learn more about the role of stromal cells in cancer progression. Several systems have been used to understand the effects of intercellular interactions with stromal cells, as well as the effects of paracrine secretions on cancer cell invasion and metastasis. Double layered alginate hydrogels seeded with prostate cancer cells and normal prostate fibroblasts in different compartments are used to study paracrine effects on shedding of E-cadherin by cancer cells and how it relates to cell-cell detachment and the initiation of cancer metastasis [92]. 

The works reviewed in this section demonstrate some of the advantages that the introduction of biomaterials into 3D cultures provides, as we search for better ways to study and treat cancer. Collaborative, interdisciplinary efforts by tissue engineers and biologists can lead to *in vitro* systems that recapitulate the different stages of cancer progression. Such systems can transform our understanding of cancer biology and aid the drug discovery and development processes.

## 4. Regulating the Tumor Microenvironment with Biomaterials for Treatment

The role of the microenvironment in the maintenance and progression of tumors makes it a relevant target for cancer treatment [93,94]. As such, drug delivery systems, predominantly nanoparticles (NPs), have been designed to deliver therapeutics that modify the microenvironment (Table 2). Polymeric NPs made from natural biodegradable materials, like chitosan and hyaluronan, are among those commonly used for the delivery of chemotherapeutics to tumors [95,96]. Synthetic polymers, like PLGA, provide versatility through the ability to modulate the release kinetics of drugs. The rate of drug release from PLGA, for instance, can be controlled by varying the amount of lactic and glycolic acid, as well as the molecular weight of the polymer used in formulation [97]. Inorganic non-degradable NPs synthesized from gold and silica are also used in the delivery of small molecule chemotherapeutics and nucleic acids [98,99]. For the delivery of nucleic acids, different polymers have been developed that complex with DNA [100,101]. For example, natural polymers, like chitosan, which is cationic, can complex with nucleic acids for delivery to tumors [102]. 

**Figure 2:**
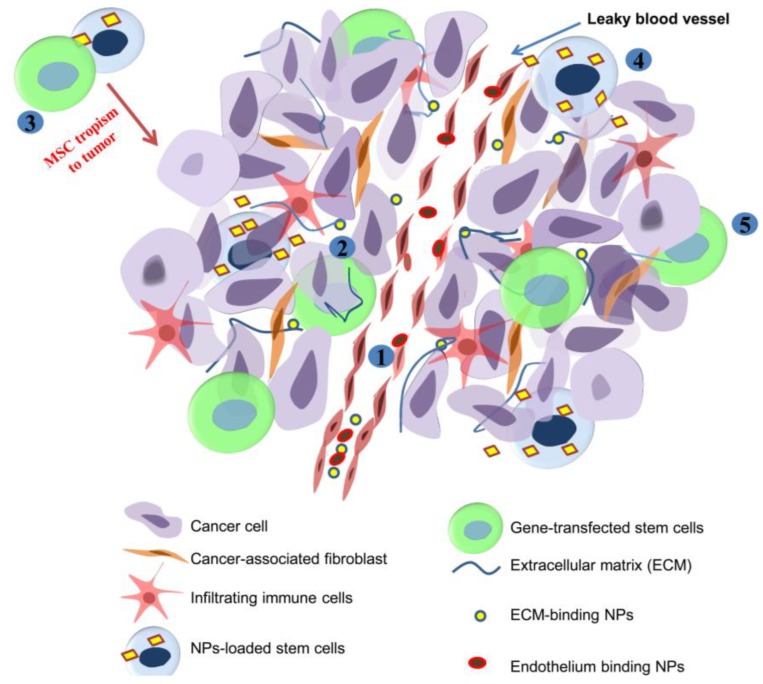
Illustration of the strategies for modulating tumor stroma for therapy. NPs injected intravenously can interact with blood vessel endothelium (1) or extravasate into the tumor stroma through the leaky tumor vasculature and bind to the ECM (2). For other applications, MSC tropism towards tumor can be used to modify the tumor stroma or induce apoptosis in cancer cells (3). MSC can be loaded with NPs that are released within tumors (4) or transformed to secrete proteins that inhibit tumor growth (5).

In addition to targeting therapeutics to tumors, most drug delivery systems provide stability, controlled release within the therapeutic window of drugs/biologics and decrease toxicity [7]. In addition to drug delivery systems, the tropism of MSCs to tumors is being leveraged to modulate the stroma and for drug delivery (Figure 2). 

**Table 2:** List of some nanotherapeutics at different stages of development.

Drug name	Nanomaterial	Therapeutic	State of development
Doxil	Liposome	Doxorubicin	Approved (US, 1995; EU, 1996)
DaunoXome^®^	Liposome	Daunorubicin citrate	Approved (US, 1996)
Feridex	Dextran coated superparamagnetic iron oxide nanoparticles (SPION)	–	Approved (US, 1996)
Myocet	Liposome	Doxorubicin	Approved (EU, 2000)
Abraxane	Albumin NPs	Paclitaxel	Approved (US, 2005; EU, 2006)
Genexol-PM	PEG-PLA Micelle NPs	Paclitaxel	Approved (South Korea, 2007) Phase III trials
Lipoplatin	Liposome	Cisplatin	Phase III trials
OPAXIO	Polymer-drug conjugate	Paclitaxel	Phase III trials
Clariscan	SPION	–	Phase III trials
ABI-008	Albumin NPs	Docetaxel	Phase II trials
AP5250	Polymer-drug conjugate	Carboplatine platinate	Phase II trials
CRLX101	Polymeric NPs	Camptothecin	Phase II trials
MBP-426	liposome	Oxaliplatin	Phase II trials
BIND-014	Targeted polymeric NPs	Docetaxel	Phase I trials
MAG-CPT	Polymer-drug conjugate	Camptothecin	Phase I trial

### 4.1. Drug Delivery to Tumor Stroma

Increasingly, attention has been directed towards targeting therapeutics to the tumor stroma in order to create an unfavorable environment for tumor progression. In some cases, such as pancreatic cancers, which have dense stroma, the stromal cells are more accessible for targeting therapeutics. The stromal cells are also more genetically stable than cancer cells and, therefore, less likely to acquire drug resistance [103].

Targeting endothelial cells for cancer treatment is an inviting strategy, since these cells form the blood vessels that support tumor growth [14,15]. Endothelial cells in tumor blood vessels express unique integrins, proteoglycans and proteases that can be used to selectively deliver therapeutics to tumors [104]. Phage display experiments have produced several peptides that selectively interact with tumor blood vessels and improve active targeting of NPs to tumors [105]. Even without conjugation to NPs, some tumor blood vessel-targeted peptides are able to increase the amount of drug that accumulates within tumors. Such peptides work by activation of integrins on endothelial cells, which help transport small molecule drugs into tumors [106]. Aptamers that selectively bind to tumor vasculature, such as nucleolin binding aptamers, target drug-loaded NPs to endothelial cells in gliomas and result in better tumor inhibition than untargeted NPs [107]. Other targeting ligands used for drug delivery to tumor vasculature include antibodies to VEGFR-1, VEGFR-2 and α_v_β_3_ integrin, which are overexpressed on the blood vessel endothelium [7]. Because blood vessels are easier to access than cancer cells, targeted drug delivery to the tumor blood vessels can increase the amount of drug present in tumors. 

In another strategy, drugs are delivered to the ECM rather than the blood vessels, by utilizing the enhanced permeability and retention (EPR) of macromolecules within tumors. The EPR effect, whereby macromolecules accumulate in tumors, is attributed to the leaky nature of tumor blood vessels and inefficient lymphatic drainage [61]. The EPR effect has been the foundation for most passive targeting of NPs to tumors. To improve NP retention in tumor ECM, active targeting to receptors and macromolecules in the tumor stroma is used. Hyaluronan (HA), a polysaccharide abundant in the tumor stroma, is used to increase drug accumulation [108]. The efficiency of HA at delivering drugs to tumor ECM has resulted in the development of several HA-drug conjugates, including that for paclitaxel, butyric acid and small interfering RNA [109]. NPs are also targeted actively to MMPs, such as the membrane type 1 MMP (MT1-MMP) [110] through conjugation with the Fab fragments of anti-MT1-MMP antibodies [111]. Because MMPs have roles in metastasis, they can be targeted for active drug delivery to advance-staged cancers.

### 4.2. Biomaterial-Mediated Modulation of Tumor Immune Components 

Several strategies have been developed to induce the immune system to “reject” tumors. One method involves delivering cytokines and other factors to the tumor to overturn the immunosuppressive environment [112]. With this strategy, IFN-γ has been delivered to tumors via adsorption to dimercaptosuccinic acid-coated magnetic (DMCM) NPs, to induce apoptosis in cancer cells and also to enhance antigen presentation by dendritic cells [113]. When iron oxide magnetic NPs, such as DMCM-NPs, are used for immune modulation, they can also serve as contrast agents for magnetic resonance imaging (MRI) to monitor tumor response [114]. Other cytokines, including IL-2, have been delivered with porous alginate/chitosan microspheres to activate cytotoxic T-lymphocytes (CTL) and improve tumor inhibition compared with free IL-2 [115]. 

Because TAMs are responsible for most of the immunosuppression observed in tumors, they have been the target of most investigations. Drug delivery to TAMs exploits cell membrane surface lectins, notably the mannose and macrophage galactose receptors [116]. Cationic dextran NPs targeted to these receptors delivered oligonucleotides to knockdown IL-10 and IL-12 receptors in TAMs *in vivo*, in order to stimulate their anti-tumor activity [117,118]. Another area of interest is the repolarization of macrophages to reactivate the immune system to recognize tumors. M2 macrophages have been reverted to the M1 phenotype with 5,6-dimethylxanthenone-4-acetic acid [119] and cytokines (IL-12 and IFN-γ) [120] and can find use in cancer treatment. Drug delivery systems that target such molecules to macrophages in tumors could activate the immune system against tumor for treatment. The challenge for developing such a drug delivery system will be to minimize off-target effects.

Another approach to activate the immune system against tumor is through the use of vaccines [112]. Tumor-specific antigens injected intradermally or subcutaneously are endocytosed by immature antigen-presenting cells, notably the Langerhans cells in the skin, which process and present them to cytotoxic T-lymphocytes (CTL) and B-cells. Immune activation in such a manner can be assisted by Fruend’s adjuvants. Nanoparticles (NPs) and microparticles (MPs) enhance immune activation [121,122] by preventing enzymatic degradation and dilution of the antigen. This increases the probability of uptake and the processing of antigens by dendritic cells (DC). As such, tumor antigens loaded into PLGA NPs are efficiently delivered to DCs and elicit a greater immune response than the antigen alone or antigen with adjuvant [123,124]. Engineered multifunctional NPs with iron oxide-zinc oxide cores also deliver cancer cell antigens effectively to DCs. In addition, their iron oxide-zinc oxide cores provide contrast for MRI, which allows monitoring of antigen delivery to DCs [8]. Antigen presentation with NPs can be further enhanced through techniques that improve endosomal escape of antigen-bearing NPs within cells. NPs formulated with pH-sensitive polymers that enhance endosomal escape result in better immunization than their non-pH-responsive counterparts [125].

To improve immunization, antigen-bearing NPs can be directed to lymph nodes, which have high DC numbers relative to skin, and can rapidly process and present antigens to T- and B-cells, which are also present within lymph nodes [126]. The efficiency of antigen delivery to lymph nodes using NPs is impacted by the size of the NPs. Ultra small NPs (25 nm) are transported more efficiently to lymph nodes than 100-nm NPs after intradermal injection [127] and are taken up by DCs in target lymph nodes [128]. Recently, an anti-tumor immune response was demonstrated without delivering vaccines or cytokines. Paclitaxel delivered by pluronic-stabilized poly (propylene sulfide) NPs to tumor-draining lymph nodes decreased the level of tumor-associated regulatory T-cells and could ultimately lead to tumor rejection [129]. 

### 4.3. Modifying Stem Cells with Biomaterials to Control Tumor Growth

MSCs have a tropism to tumors due to their inherent homing to sites of tissue injury/damage [130]. For instance, MSCs injected into lung tumor-bearing mice infiltrate the tumor nodule, but not the normal lung parenchyma. As such, they can be exploited for targeted cancer immunotherapy and chemotherapy. MSCs can be genetically modified to secrete interferon (IFN) β within the tumor stroma to reverse immune tolerance [56] or made to present TNF-related apoptosis-inducing ligand (TRAIL) on their surface to induce apoptosis in cancer cells [131].

Genetic engineering of MSCs to secrete IFN-β or express TRAIL is usually accomplished using viral gene delivery methods [132,133], which result in high transfection efficiencies, but pose a risk of oncogenic effects through insertional mutagenesis. Gene integration into the chromosome is also random and can produce non-uniform gene expression in the cell population [134]. Non-viral gene delivery systems, whereby DNA is complexed with cationic polymers, such as polylysine or polyethylenimine, can overcome some of these shortfalls of viral vectors and have been successful at transfecting MSCs. Several gene delivery systems, including novel dendrimers with hydrophilic cores and hydrophobic coronas, deliver plasmid DNA to MSCs with little cytotoxicity [135]. These dendrimers have cores made of poly(amidoamine), which complexes with DNA, while their coronas have hydrophobic chains that facilitate interaction with cell membranes to improve cellular uptake. 

Another use of stem cells is to use them as Trojan horses to deliver chemotherapeutics to tumors. MSCs loaded with drug-containing NPs migrate to tumors, where the NPs can be released from MSCs by cell membrane rupture or stimulus-induced apoptosis of the MSCs [136]. NPs can also be conjugated to cell membrane of MSCs, so that the cells survive to contribute to tumor inhibition [137]. In less vascularized tumors, like pancreatic cancer, using MSCs as Trojan horses to deliver chemotherapy can be an effective means of treatment [61,138]. 

### 4.4. Identification and Regulation of Matrix Remodeling Enzymes with Biomaterials

ECM degradation by MMPs and proteases containing a disintegrin and metalloproteinase (ADAM) affects cancer cell growth, migration and invasion. Consequently, their levels in the blood of patients can predict the extent of invasiveness [110]. Thus, methods that detect MMPs and ADAMs in tumors will help in disease diagnosis and the monitoring of the response to treatment. Techniques being developed include fluorescent activatable peptides, which contain cleavage sequences for MMP-2 and can be conjugated to nanoparticles to detect the level of the protease in tumors [98]. 

Because MMPs influence cancer progression, several MMP inhibitors (MMPI) have been developed with the hope of inhibiting metastasis. However, clinical trials of these drugs did not result in improved patient outcomes and had some adverse effects, including musculoskeletal pain and inflammation [139]. The ineffectiveness of the MMPIs was due to inefficient tumor targeting and bioavailability, while the non-specific targeting of all MMPs by MMPI accounted for the adverse effect. Problems with bioavailability and tumor targeting have been addressed by several drug delivery approaches that increase the accumulation of MMPIs into tumors and minimize non-specific tissue accumulation; for example, superparamagnetic NPs for which chlorotoxin can be bound to deactivate MMP-2 [140]. Efforts are also being made to develop inhibitors that target tumor promoting MMP-2 and -9, but are less effective on MMP-1, -7 and -11, whose inhibition causes the side effects. 

Biomaterials by themselves have also been shown to inhibit the production of MMPs. Gadolinium metallofullerenol (GMF) NPs, for instance, inhibit the production of MMP-2 and -9 with a resultant decrease in tumor invasiveness. Although the mechanism through which GMF NPs inhibit MMP is unknown, tumors in animals treated with GMF NPs were encased in fibrous tissue, which minimized metastasis [141]. 

## 5. Future Perspective

Tissue engineering continues to provide ways to model different aspects of cancer and has played crucial roles in understanding the disease. With new biomaterials and 3D *in vitro* models that mimic some of the biological, chemical and mechanical properties of tumors, new insights into cancer initiation and progression are being acquired. Further development of these systems will lead to recapitulation of the complex architecture, cellular hierarchy and mechanical and fluidic properties of tumors, which inherently direct cell-cell and cell-ECM interactions. Another area of future studies is modeling of the dynamics of the evolution of tumors from ignition to metastasis. Current *in vitro* systems are static and only give “snapshots” in disease initiation and progression. *In vitro* models that are able to copy the evolution and transitions of tumors over the disease period in response to different stimuli will significantly improve our understanding of the disease process and guide the development of better treatment strategies. Such models could involve a hybrid microfluidic and scaffold system that fosters the continuously changing interactions between different cell populations in the tumor. 

Another area that requires further research is the creation of high-throughput 3D *in vitro* tumors that model different stages of cancer for use in drug discovery and development. Current high-throughput 3D tumor models, which are mostly spheroids, are not useful for screening of drugs that could potentially prevent or treat metastasis, thus making the discovery process laborious, time consuming and expensive. 

Cancer immunotherapy is a growing field and stands to benefit from advances in biomaterials. Biomaterials that independently activate the immune system against tumors or deliver therapeutics that perform that function can improve the treatment of advance-staged cancers. Biomaterials, formulated as NPs and MPs, could also serve as platforms for the rapidly advancing field of cancer immunization to prevent cancer initiation altogether. 

## 6. Conclusion

Control of tumor progression by modulating the activity of the tumor stroma holds promise. Biomaterials provide ways to target therapeutics to the tumor microenvironment to reprogram different cells in the stroma. As we gain more understanding of tumor biology, the role of the microenvironment is expected to take center stage in strategies to control tumor initiation, progression and metastasis; and biomaterials will play an integral role as we aim to alter the stroma to make it less favorable for cancer progression. 
